# Hot topics, urgent priorities, and ensuring success for racial/ethnic minority young investigators in academic pediatrics

**DOI:** 10.1186/s12939-016-0494-6

**Published:** 2016-12-09

**Authors:** Glenn Flores, Fernando S. Mendoza, Elena Fuentes-Afflick, Jason A. Mendoza, Lee Pachter, Juan Espinoza, Cristina R. Fernandez, Danielle D. P. Arnold, Nicole M. Brown, Kymberly M. Gonzalez, Cynthia Lopez, Mikah C. Owen, Kenya M. Parks, Kimberly L. Reynolds, Christopher J. Russell

**Affiliations:** 1 Medica Research Institute, Mayo Clinic, and University of Minnesota School of Public Health, MR-CW105, P.O. Box 9310, Minneapolis, MN 55440 USA; 2 Stanford University School of Medicine, Medical School Office Bldg., X240, 1265 Welch Road, Stanford, CA 94305 USA; 3 University of California, San Francisco, School of Medicine, 1001 Potrero Ave, SFGH 5, San Francisco, CA 94110 USA; 4 University of Washington School of Medicine, and Seattle Children’s Research Institute, Suite 400, M/S: CW8-6, PO Box 5371, Seattle, WA 98145 USA; 5 Drexel University School of Medicine and St. Christopher’s Hospital for Children, 160 East Erie Ave, Philadelphia, PA 19134 USA; 6 Children’s Hospital of Los Angeles and the Keck School of Medicine, University of Southern California, 4650 Sunset Blvd., MS 76, Los Angeles, CA 90027 USA; 7 Columbia University Medical Center, 630 West 168th Street, Presbyterian Bldg 17-201I, New York, NY 10032 USA; 8 University of Colorado School of Medicine, 13123 E. 16th Ave, B-158, Aurora, CO 80045 USA; 9 The Children’s Hospital of Montefiore, 3444 Kossuth Ave, 1st Floor, Bronx, NY 10467 USA; 10 Robert Wood Johnson Foundation Clinical Scholars Program, University of Pennsylvania, 13th Floor, Blockley Hall, 423 Guardian Drive, Philadelphia, PA 19104 USA; 11 University of Texas Health Science Center at San Antonio, 7703 Floyd Curl Dr., MSC 7808, San Antonio, TX 78229 USA; 12 University of Florida College of Medicine, 841 Prudential Dr, Suite 1330, Jacksonville, FL 32207 USA; 13 University of Texas Medical School at Houston, 6431 Fannin St., JJL495, Houston, TX 77030 USA; 14 University of Miami Miller School of Medicine, 1611 NW 12th Ave, Miami, FL 33136 USA

**Keywords:** Workforce, Diversity, Minority groups, Racism, Discrimination, African Americans, Hispanic Americans

## Abstract

**Background:**

The number of racial/ethnic minority children will exceed the number of white children in the USA by 2018. Although 38% of Americans are minorities, only 12% of pediatricians, 5% of medical-school faculty, and 3% of medical-school professors are minorities. Furthermore, only 5% of all R01 applications for National Institutes of Health grants are from African-American, Latino, and American Indian investigators. Prompted by the persistent lack of diversity in the pediatric and biomedical research workforces, the Academic Pediatric Association Research in Academic Pediatrics Initiative on Diversity (RAPID) was initiated in 2012. RAPID targets applicants who are members of an underrepresented minority group (URM), disabled, or from a socially, culturally, economically, or educationally disadvantaged background. The program, which consists of both a research project and career and leadership development activities, includes an annual career-development and leadership conference which is open to any resident, fellow, or junior faculty member from an URM, disabled, or disadvantaged background who is interested in a career in academic general pediatrics.

**Methods:**

As part of the annual RAPID conference, a Hot Topic Session is held in which the young investigators spend several hours developing a list of hot topics on the most useful faculty and career-development issues. These hot topics are then posed in the form of six “burning questions” to the RAPID National Advisory Committee (comprised of accomplished, nationally recognized senior investigators who are seasoned mentors), the RAPID Director and Co-Director, and the keynote speaker.

**Results/conclusions:**

The six compelling questions posed by the 10 young investigators—along with the responses of the senior conference leadership—provide a unique resource and “survival guide” for ensuring the academic success and optimal career development of young investigators in academic pediatrics from diverse backgrounds. A rich conversation ensued on the topics addressed, consisting of negotiating for protected research time, career trajectories as academic institutions move away from an emphasis on tenure-track positions, how “non-academic” products fit into career development, racism and discrimination in academic medicine and how to address them, coping with isolation as a minority faculty member, and how best to mentor the next generation of academic physicians.

## Background

Recent US Census data document that racial/ethnic minorities comprise the majority of the population in 370 US counties and the District of Columbia, and that the population growth of Latinos, Asians, and African-Americans is far outpacing that of whites in America [1]. Projections indicate that the number of minority children will exceed the number of white children in the US by 2018 [2]. Nevertheless, although 38% of Americans are minorities [3], only 12% of US pediatricians [4], 5% of US medical-school faculty [5], and 3% of US medical-school professors [5] are minorities. Furthermore, only 5% of all R01 applications for National Institutes of Health (NIH) grants are from African-American, Latino, and American Indian investigators, and African-American investigators are significantly less likely than white investigators to be awarded an NIH R01 (Research Project Grant), even after adjustment for relevant covariates [6].

Prompted by the persistent lack of diversity in the pediatric and biomedical research workforces, the Academic Pediatric Association (APA) Research in Academic Pediatrics Initiative on Diversity (RAPID) was initiated in 2012, with support from a Professional Society Programs to Promote Diversity (R25) grant from the National Institute of Diabetes and Digestive and Kidney Diseases (NIDDK). RAPID targets applicants who are members of an underrepresented minority group (URM), disabled, or from a socially, culturally, economically, or educationally disadvantaged background. The program, which includes both a research project and career and leadership development activities, consists of: 1) small research grants in NIDDK mission areas (pediatric obesity, nutrition, and/or sickle cell disease); 2) pairing of the RAPID scholar with a national mentor who is an accomplished senior investigator and seasoned mentor; 3) RAPID Scholar telephone conference calls every two months, to provide peer support, peer mentoring, networking, a venue for presenting research in progress, and opportunities for potential research collaborations; 4) in-person mentoring and networking at an annual breakfast at the Pediatric Academic Societies (PAS) meeting; and 5) an annual fall conference on career development, leadership, and academic success which is open to any resident, fellow, or junior faculty member from an URM, disabled, or disadvantaged background who is interested in pursuing an academic career in general pediatrics [7].

The aim of this paper is to identify hot topics, urgent priorities, and how to ensure success for racial/ethnic minority young investigators in academic pediatrics, based on a special session which occurred at the 2015 RAPID Conference. No previous published work, to our knowledge, has ever addressed these issues using the innovative approach of having young investigators identify such “hot topics,” and then addressing these critical issues by posing questions to a panel of nationally renowned senior academicians from diverse backgrounds.

## Methods

As part of the annual RAPID fall conference, a Hot Topic Session is held in which the young investigators spend several hours developing a list of hot topics on the most useful faculty and career-development issues. These hot topics are then posed in the form of six “burning questions” to the RAPID National Advisory Committee (comprised of eight accomplished, nationally recognized senior investigators who are seasoned mentors, three of whom attended the conference, and two of whom were present for the panel), the RAPID Director (GF) and Co-Director (FSM), and the keynote speaker (LP).

The 10 young investigators who attended the 2015 RAPID fall conference hailed from institutions across the US and included the two 2015 RAPID Scholars (JE, CF), five assistant professors, two fellows, and one chief resident, of whom seven are African-American and three are Latino. The six compelling questions posed by these young investigators—along with the responses of the senior conference leadership—provide a unique resource and “survival guide” for ensuring the academic success and optimal career development of young investigators in academic medicine from diverse backgrounds.

## Results: dialogue and discussion

Topics identified by the young investigators and addressed the senior conference leadership include negotiating for protected research time, career trajectories as academic institutions move away from an emphasis on tenure-track positions, how “non-academic” products fit into career development, racism and discrimination in academic medicine and how to address them, coping with isolation as a minority faculty member, and how best to mentor the next generation of academic physicians. The text that follows is a verbatim transcript of the session held during the 2015 RAPID fall conference (with minor editing for clarity and brevity).

### Negotiating for protected research time

**Mikah Owen:** How do we negotiate for more protected research time in our jobs for whatever area of interest we may have?

**Jason Mendoza (JM):** When you’re negotiating in general, just remember to negotiate. Remember to let them know what you want, because one of the things, especially when you negotiate with your own institution, is that you forget that they may not have your best interests at heart. They have to look out for their division or department, and so you need to really set out, “this is what I need and this is why.” Have protected research time, a timeline for your K [NIH mentored career-development award], people who are going to be your mentors on board and who can back you up in other conversations. It’s a negotiation, and you need to put out really concrete steps so that the division head can picture your progress, see where you’re going. And the timeline which you’ll get, it’s usually, hopefully, within two years of you getting there to include time for K revision and resubmission. I think just having that laid out for them will be good.

**Elena Fuentes-Afflick (EFA)**: Having seen both sides of this issue, being intentional about it, as Jay [Mendoza] is mentioning, is really important. There are some really helpful books about negotiation and I would encourage you to read them. It’s a mistake to say, “I want this, I want that, etc.” As Jay’s mentioned, you have to understand the context of your colleagues within the division and department. It’s fine to advocate for yourself, but remember that there are other factors, it’s not just a one-off kind of decision. And also try to think about the person from whom you’re making the ask. What are the things that they want in terms of success, their division, for your clinic, for your department? How can you speak the language that they want to hear, and somehow, by them helping you, you are helping them, and then it can become a virtuous cycle where, yes, it’s an investment, but the return is helping them advance another kind of agenda. And those are examples of the kinds of tips that you can learn from books. There’s, of course, *Getting to Yes*, and other ones. There are also workshops that you can attend. I keep plugging PAS workshops, but there are some really helpful little tips that you can incorporate. I encourage you to rehearse, because Glenn [Flores] was making the point about the common use of “um” and “ah.” The more that you stumble over something that you’re saying, it communicates to the other person, “I’m feeling uncomfortable about this, that’s why it’s hard for me to get it out.” But if you rehearse, it won’t come across too strong, because an ultimatum is unlikely to help you. If you’re going to make an ultimatum, be sure that you are ready to follow through, and very rarely will you be ready to do that. Make sure to speak in a respectful and constructive way, but it’s fine to advocate and self-promote, just don’t come across too strong, and sometimes, rehearsing with a colleague or your mentor can really help you with that.

**Glenn Flores (GF):** Address two key issues: one is logic and the other is return on investment. Explain to your supervisor why it’s important for you to have the protected time. For example, you could say, “I need to develop my research portfolio, and this will reflect well on the division and department if I get publications and grants.” The return on investment is those products. There was a time when any kind of clinician-investigator position was a standard of at least three years of 50–75% protected time for research, along with research seed funds. Those days seem to be either gone or going by the wayside at a lot of institutions, so you have to get an idea of what’s the pulse at the institution that you’re thinking about, because there are some institutions where you have to come out of your fellowship with a K Award already in hand. Unfortunately, a lot of institutions are moving in that direction, so that is a whole another structured way of thinking. So that’s a third key issue: assess the pulse regarding what’s the industry standard at your institution, and then you’ll be in good shape.

**Fernando Mendoza (FM):** I would just add the issue of “show me.” As a division chief, I don’t know everything everybody does. You need to come with the presentation, “let me show you what I’ve done and what I’ve accomplished, and here’s what I need to continue this work. When you do negotiate, it may not be a one-time process, but rather, you should be ready to come back and say, “you gave me the opportunity to do research and I got this research project done, I got this paper published,” etc., “so now I would like continued support for X.” At the end of the day, the negotiation that everybody talks about is really relationship building, and that relationship has to be based on trust, and the trust has to be based on positive outcomes, outcomes that people want done. If I support research time, and nothing happens, then when they comes back to me again and say they want more research time, I’m going to have to say, “well, what happened last time?” So, think about negotiations at any level as trying to create the relationship of trust, positive outcomes, and adding to what your chief wants and what you want.

**JM:** Can I just add one thing? If you’re considering more than one institution, it can be helpful to let them know about that. When people are competing for you, it can give you a little bit of leverage and actually show how you’re valued outside of just one institution.

**GF:** Four of the five of us [on the panel] have been or are division chiefs, and I don’t know if Lee wants to weigh in on this, but you have to understand what’s going on in the mind of the person you’re asking for the protected time. These days, it’s a combination of making sure you’re maximizing RVUs [relative value units, a metric for value used in US Medicare reimbursement formulae for physician services and procedures], making sure you’re getting all the teaching done that you’re supposed to get done, and keeping your department chair happy, who’s usually pressuring you to get those RVUs. And now, we’re seeing that increase. What even constitutes a full-time clinician is up for debate, because it used to be 80% clinical effort, and then you have the one day of administrative time, but now, we’re seeing institutions requiring 90% or even 100% clinical effort, so it’s starting to evolve into a completely different landscape, and it’s important for you to understand the different pressures and dimensions.

### Career trajectories as academic institutions move away from emphasizing tenure

**Kymberly Gonzalez:** In an environment moving away from the typical tenure track, how will our careers be different than the environment before?

**EFA:** I’m going to try to answer wearing my academic affairs hat, because I interact with faculty who are basic scientists, clinicians, everyone, and I think that we have mythologized what an academic career used to be in terms of the benchmark that we’re using for comparisons. There’s no question that as money has become tighter, there are new realities, but I think that the past was not quite as rosy as we think it was, and now we have to demonstrate our contributions in all mission areas, which means that you need to consider your clinical contributions, education, scholarship, and service. As we look to the future, I believe we’re going to see some new structures. We’re going to be less departmentally or divisionally restricted, because we’ve had very strong silos between our organizational units. I think those are going to change, but that’s going to take a while to change, especially the departmental ones. We’re very wed to our departments. If you’re a generalist, by definition, some of those criteria don’t adequately represent our clinical value, so I think we have the opportunity to define value on our own terms. Institutions are rooted in history and it’s going to take a while, but we need to look at the future as an opportunity to define ourselves and our rules in ways that we want to define, rather than fitting into the existing structure. We’ve been discussing tenure, what it means and whether we should we have it. That conversation will last another 20 years and will undoubtedly affect all of you, depending upon what kind of career path you choose. The other elements of academic life will remain largely in place, but I still think it’s important for you to decide how you want to add value to your institution and how you want to define value.

**Lee Pachter (LP):** Each institution is very different. I’ve never been on a tenure track in my whole career. If you’re working at an institution which is actually a medical school, tenure track is an important thing, but when I was in Connecticut, I was working at a teaching hospital, so my payor was actually the hospital, and not the university, so tenure was not an issue. Then, when I came to St. Christopher’s, I’m a Drexel faculty member, but Drexel doesn’t pay me, St. Christopher’s pays me, so I’ve never been on a tenure track, and I think that there are some traditional institutions where tenure is really important. But for many of the places that you’re going to be looking for positions, tenure isn’t even going to be an issue.

**GF:** I would add that tenure is not what it used to be. Customarily, based on the traditional European system, it was supposed to allow you liberty to pursue intellectual interests and the security to do so. Now, it’s such a rare bird in the US. Some institutions have abolished it. At a lot of institutions, you can get tenure, but in some senses, it’s meaningless. For example, I know of at least one institution where you get tenure, but you have an annual contract that gets renewed, and so, theoretically, you could either be let go, or you could be put into a traditional university position (instead of a school of medicine position) where your salary would be severely cut. Thus, there are a lot of ways that institutions can “extract” you from their faculty, if they want to, so it doesn’t have the same meaning that it used to. Now, on the flip side, there are still institutions where it’s an up-or-out situation where you have to get promoted to the next level, or you’re gone or get knocked down to a position that you wouldn’t want to be knocked down to. So you really have to be aware if you’re applying for a job in which one of those systems is in place. But it really is no longer, in most cases, a situation where you’re there for life and you don’t have to worry. Even in places where there’s more security, there still is tenure review, in which the chair sends a letter to your dean stating whether you are “where you should be.” So, I just wanted to open your eyes, because I had to learn that in the past few years, and it’s a constantly shifting changing landscape.

**FM:** I would add that what you’re hearing is the effect of money. And one of the things you ought to do is look at your institution and see how the money flows. Many institutions have gotten rid of tenure because that was a contract that made them pay salaries for people that may not be achieving the expected productivity. More and more, I think it’s more difficult to get tenured, because you have to be able to show that you can be productive in grant writing, and supporting your salary. Now that getting NIH grants is more difficult, schools are reluctant to invest in that area without some assurance. So, in general, most schools will have three academic lines. One is a “tenure line,” which usually means that you are primarily doing about 80% research, and the assumption is you’re going to be able to support yourself from grants. The second line is the clinical scholar. You do clinical work that can range from 50 to 80%, along with 20–50% research. And then the clinician line or educator line, in which you primarily see patients, teach, and you may do some scholarship. There’s variability within these lines among schools, depending on what their traditions are with respect to faculty lines, and how much money they have to support scholarship. This is not to scare you, just to make you aware about faculty lines.

**GF:** I’ll add one thing that didn’t occur to me until a little bit later in my career, which is probably more meaningful now if you can get it, and that depends on you, obviously, being highly productive and well-recognized: an endowed professorship or endowed chair. I imagine a lot of you don’t necessarily understand what that is, but it is essentially a donor makes a big contribution, let’s say it’s a million dollars. You then get the interest from the principal every year. So if it’s for a million dollars, at a typical 5% return on the interest, that’s $50,000 a year. There are even bigger endowed professorships and distinguished chairs. So that’s, for me, the Holy Grail: when you get one of those, you have this nice money to play with and you can use it for essentially anything that’s research related. You can use it for trainees, your own research, or a statistician, so it’s really nice, it’s something eventually to think about, but at the beginning of your career, you may not even be aware of it, other than thinking it’s an honorific.

**FM:** People often use that for their salaries, too.

**GF:** That’s the other good thing. You could support your salary with an endowment so that you don’t have to be in clinic.

### How “non-academic” products fit into career development

**Nicole Brown:** We were interested in your thoughts about nonacademic products, like op-ed pieces, policy briefs, and white papers, and how they’re perceived in a career-development trajectory.

**EFA:** I review hundreds of CVs every year in my academic affairs role, and I think what matters relates to the theme that Glenn mentioned about endowed chairs. Institutions have a lot of specific rules, and some rules are universal, but you need to ask questions about your institution, the criteria for advancement. At my institution, we have five titles, and each title has slightly different expectations, so you need to know by what measure you will be judged. For some of our titles, pieces like op-eds or editorials, especially in professional journals, can be very important, although, usually, you have to be standing on a body of accomplishment to be asked to write an editorial, which is a more of a thought piece. Most institutions want original scholarship, whether it’s a case series which demonstrates some new clinical entity, or the more traditional research publication. In our CV structure, we have peer-reviewed publications and then other publications.

**GF:** I would emphasize that there’s what you do which is going to be considered for tenure, and there’s what you want to do in your career, and op-eds are a great way to call attention to important policy issues. I’ve published a few, and some of them have really had some nice impact, and in ways I didn’t anticipate. So I would encourage you to think about them. There is one big caveat: you need to be careful about your affiliation when you publish your op-ed. We had some issues at a former institution about people listing their affiliation at a state university, and, essentially, the op-ed potentially coming across as a “state-sanctioned” political viewpoint. That can cause a lot of trouble, and institutions may be very explicit about prohibiting this. There are ways to work around it. You can list yourself simply as “a pediatrician,” or if you have a hospital affiliation and your hospital is comfortable with it, you can list the hospital affiliation, but you really have to vet what you can put down, because it is a form of lobbying in some states universities, and can actually invoke some legal issues.

There’s a fixed part of the promotion/tenure process where there’s a committee, and they’re going to have their criteria, and that process may not necessarily change, but the part that you may not know about is, it usually varies from institution to institution, but your division chief is the one who puts you up for promotion, so you need to work with your division chief to put together your package and make the arguments on your behalf. Therefore, if you’re saying that you’re a policy expert, op-eds and issue briefs would be really important, and maybe something that the promotion/tenure committee, even though they don’t normally weigh heavily, may say, “okay, in their realm, this is important.” I don’t think there’s any single correct answer to your qeustion, but as Elena was saying, there are some traditional products that people look at and sometimes will weigh more heavily.

**LP:** If you take out the whole issue of tenure and promotion, just in terms of the research that you’re doing, it depends. These non-academic pieces can be both professional journals or in the lay media, and if you’re an advocate and working in the community, having your name in the press is actually really helpful, because it gives you name and credibility in the community. It might open up some doors in the community. In terms of the academic stuff, let’s say it’s a non-peer-reviewed piece. When you get evaluated, you get evaluated on your research, your education, your clinical, but there’s also something called professional leadership. So if you do an editorial or something that may not be peer-reviewed, but shows that you’re a thought leader, that goes into you being evaluated in terms of your professional leadership.

**FM:** I would just add that this comes back to the issue of what does it take to get promoted at your school in your line. So your homework is to figure this out, because it may be that these kinds of things are not useful for your promotion. We want to leave you with the idea that it is important to know, and be conscious about whether an activity is going to help you or not. This is important because a lot of people do activities that don’t always help them. So, when you go back to your institution and figure out what exactly it takes for you to get promoted, it should be your informed judgement of where you think an activity has academic value. Finally, there are a lot of things happening in communities right now that you may want to speak about, and that’s great, but it may not necessarily be part of your CV.

### Racism and discrimination in academic medicine and how to address them

**Christopher Russell:** We wanted to hear a little bit about if any of you on the panel had experienced racial or other types of discrimination in your career and how you dealt with it. Do you think that racial and other biases are different in the workplace now, better, or different, and what we can do to change it?

**EFA:** I’ve experienced gender discrimination. When I was interviewing for a job, I was pregnant with my second son, and the interviewer, an older man, knew that I was pregnant. He told me something like, “it’s very difficult to be a mother and an academic.” He didn’t say, “I don’t know if you can succeed,” but that was, of course, right there, and I was rendered speechless, because I had not raised the issue of my pregnancy. I didn’t know what to do, because I thought, I’m probably not going to get this job, since he raised the issue, and I didn’t tell him, “you have no right to say that.” I was very upset, and wrote a follow-up letter that didn’t directly address the pregnancy, but stated that I was aware of the expectations, and reiterated my qualifications and interest. I got the job, and we can call that a happy ending. But what if he hadn’t said it, but had the thought and had acted on it? Now, with more publicity about the laws and rules and the antidiscrimination clauses, I hope that none of you hears anything like that. We now have all kinds of reporting requirements, and Title IX offices, all of which can trigger investigations, if that kind of thing ever happens. Every institution has to have a mechanism to handle these issues, so, if it happens to you, please report it to the appropriate person. You can report it anonymously, but institutions have an obligation to respond. They are also supposed to educate people about what you can and cannot ask; however, if you raise a personal issue, then it’s okay to discuss. For example, if you ask whether there is a childcare center, you’ve raised the issue of children. I’m not saying you shouldn’t, but just understand that, sometimes, these issues can be very tricky.

**FM:** In my career, there have been episodes. One was with a patient early in my career. There was a patient I had; the mother said, “you know, I don’t want to be treated by a Mexican doctor.” I said, “fine, let me find you another doctor,” and felt that the patient was clearly racist, but, for me, the situation was, why should I carry her burden of being racist? I said to myself, “well that’s your problem. I’m concerned about your child and my job is to do the best for your child, so let’s get you a doctor that you feel comfortable with.” I don’t need to deal with people that are racist. I always felt that I can’t stop people from being racist, but I can stop how it affects me. I go back to what my core values are, which are treating people fairly and compassionately. For some people, it’s kind of hard to do, but I also have the core value of a pediatrician having my primary concern for the child. So if it was a child abuse issue, I would not have been flexible, But, in this scenario, it was a child with asthma, so I was fine with linking her with another physician, but I think it’s important for me to have control of the situation. We always try to make our environment less with the “isms,” such as racism, but it’s hard to do. We’re not seeing a lot of change in the last 20–30 years, but I do think that we need to figure out for ourselves, as leaders, how we deal with it, how it affects us, and how we can help trainees to deal with it.

Whatever we do, we can’t take people’s problems or their racism and put it on our shoulders. We have enough issues in our own personal lives. So you have to find a way to say, “that’s their problem, if they start affecting me or my colleagues, then I need to do something about it.” Racism and other “isms” are issues that all of us have to be active in addressing and pushing the envelope to make sure it doesn’t affect the care of our patients. But as we do this, we also don’t want to take on the burden solely by ourselves.

**GF:** I can relate two experiences. One was personal, in terms of promotion and tenure, and I want to say that, these days, it’s more insidious. I don’t think it’s quite as overt as Dr. Mendoza might have experienced many years ago, but I think it’s just as intense in terms of the magnitude. Early on in my career, when I was up for promotion from Assistant to Associate Professor, I thought that I had an excellent track record in what, at that time, was the burgeoning field of disparities. I had published a fair amount in major journals and had several substantial grants and thought that I was worthy of promotion. It was my fifth year as an assistant professor and I didn’t get promoted; I didn’t feel I was advocated for in the process and that my work wasn’t valued. I felt that it was due to some racism, and so I subsequently vigorously advocated for myself, including pointedly mentioning that maybe I didn’t get promoted because of the area (disparities) or my “background” (being Latino). And the next year, I did get promoted. So that’s how I dealt with it, but I’ll never really know for sure what happened, and it concerned me, particularly because I believe I was the first minority investigator faculty member ever up for promotion in the Department.

And then the other issue is that it is systemic, and done in such an insidious way that you may not necessarily think it’s racism or bias. For example, I was at another institution where they had a faculty conference with all 200 faculty there, and they said, “we need to enhance our case mix because our profit and our revenues are lacking. So we’re now going to limit the percent of Medicaid covered kids that we’ll see.” And, in particular, they were talking about doing this in clinics that were located in more affluent neighborhoods. I stood up in the middle of this faculty conference and said, “this really bothers me. This is actually sounding like a form of discrimination.” I then shared the famous MLK quote: “Of all the forms of inequality, injustice in health care is the most shocking and inhumane.” People looked astounded, and I actually got some good feedback later on about it. They backed off of this “initiative,” but I felt that it was part of a systemic approach to racism and discrimination against low-income and minority children. The other thing going on was that a lot of the undocumented kids were being seen in federally qualified health centers, and they had chronic diseases, and they clearly needed specialty care for some of the most severe conditions, such as renal failure. And yet, this major children’s’ hospital was making it *harder*, just outright refusing to see patients who either didn’t have insurance or were on Medicaid, so that the primary-care physicians and pediatric residents at the federally qualified health center had to essentially be the subspecialists. I raised this a number of times and didn’t get all that much traction. But sometimes, it’s essential for *you* to be the voice to alert people to the fact that what they’re proposing or doing may ostensibly sound like it’s helping in terms of the finances, but it’s actually not a good population-health approach and can easily be seen as systemic, systematic racism and bias.

**FM:** Let me just add one more thing. It’s important for you, at your stage, to find somebody that you can talk to about these things, too. The hardest thing is just to have it internally. It may be your colleagues, it may be senior deans, but this is going to happen, and you just need to make sure that you have somebody to talk to. And, at the end of day, the thing that we want to do through this RAPID conference is create resiliency and success, as these are burdens that can really hinder things. And if you can find a way to deal with it internally, but also externally with colleagues and the appropriate administrators, that’s a real positive thing. It’s a great buffer.

**GF:** I want to echo what Fernando said, too, because there are studies, putting on my scientist hat, really impressive studies by Jim Collins about maternal lifetime exposure to interpersonal racism, and that it’s strongly associated with very low birth weight in infants and preterm delivery, but active coping behaviors weakened this relationship. What Fernando brought up is important. You’re going to experience racism and discrimination, and you may already have been victimized by them, so you need to talk to your colleagues and to a trusted mentor so that this doesn’t end up taking a toll on you, but rather allows you to be resilient and to do something positive in response.

### Coping with isolation as a minority faculty member

**Kimberly Reynolds:** As a follow-up question, how do you deal with some of the isolation that’s been shown in the literature that minority faculty members feel? There’s the pressure to serve and to be the “token,” but there’s also a lot of, just, “isolation.” Have any of you experienced that, and how do you deal with it?

**FM:** When I became faculty in 1981, I was the only minority faculty, but there were people who were not minority that were very supportive, so you’ve got your friends, the people who are always there for you, and then as we grew, other people, minority folk, came onto the faculty. You have to look and see, where is your social support? Social support is probably one of the strongest things to fight and buffer stress, and, for me, it was that kind of process, finding people both internal and external to the medical school. I was the only minority in the whole pediatrics department, so I looked throughout the med school and looked outside. This resulted in my main mentor being an anthropologist, a Latino. It’s extra work, but the reward is having somebody that you can connect with.

**Juan Espinoza:** Also as a follow-up, my dad was a career NIH scientist. He was there for 20 years, and he always told me, from the time that I was in high school, do not apply for anything that says “minority” in it, because it may change the way people perceive you. It’s something that I’ve struggled with in my career, having that as the viewpoint. Looking back, what was the role of those kinds of opportunities or programs in your career? How do they reflect on us, is there an insidious underside to some of these things, and how does that balance out long-term?

**FM:** My career at Stanford has been one of supporting minorities for 33 years. And I’ve had faculty say, “I don’t want to be considered minority faculty.” Okay. I don’t see you as minority faculty. But here’s the thing: as long as we perceive ourselves to be less, we’re going to be less. I did an early pipeline program bringing students to Stanford before they started medical school. I said to them, “I want you to be faculty, and you are going to be faculty here. We want you to be a part of our Stanford family.” We have now a number of minority alumni who are faculty at Stanford and other medical schools across the country. This happened partially because we believed that they could become faculty, creating this expectation in their minds. In two years, this country will have half of all the kids being minorities. Are we still going to believe that minorities can’t become faculty? In 20 years, half of the US population will be minorities. We need to show that it’s not just being successful, it’s showing other people, “I’m a minority and I’m successful.” And that, at the end of day, is how things change.

You all can be extremely successful, but you still have to be who you are. Moreover, this helps me show students in the pipeline, here’s somebody that looks like you, and look what they’ve done. And I know, it’s easier, perhaps, not to be a “minority,” and if you don’t look a minority then it’s easier, right? But I think that that’s part of the issue; we need to say who we are and what we come from so that we can make an impression. And it’s not just really the impression for the people that don’t believe, it’s the impression for us, too. You’ve been impressed by those of us here, right? Hopefully, that makes you think that you can do it, because you can. But at the end of day, it’s going to have to be that kind of effort, the country is fighting stereotypes right now, this is what you see now and will see for the next 10 years, 20 years. We had civil rights, now we have something different: we have human rights, the ability to achieve your fullest potential. We have to believe this country is equal throughout. And that, at the end of day, is not going to be something that I do, because I’m at the tail end of my career. It’s going to be you guys.

**JM:** I wanted to add something. In talking about specific RFAs [requests for applications] and NIH grant opportunities for minorities, I would say, seize those opportunities, because the reason they exist is because whatever “normal” opportunities there are haven’t been doing a good job of being inclusive and hitting populations and topics that are important for minorities. And if you propose excellent science, and, of course, you get excellent results, no one should look on it askance. You are moving the field forward in a way that couldn’t or didn’t happen in the past, so seize those opportunities.

**LP:** White privilege has been going on for so long. Basically, what I see these programs doing is trying to balance out the issues that we’ve had for generations and generations of white privilege. So I see nothing wrong with it and I would seize the opportunities as well.

**GF:** I would encourage you to stand up and be counted. There’s some risk involved. There are the studies that show that if you submit an application for a job and your name is Shamika instead of Buffie, you’re less likely to get a job interview, and, obviously, if you have a Latino-sounding name, I would assume the same principal is in effect. But, one of the most cogent things I’ve ever heard was from a medical student who was interviewing at a residency program. She said to me, “I’ve looked at the faces of the residents and I counted how many minorities there were, and there weren’t a lot.” And *we* do that: we look at an institution and say, is this institution going to be diversity friendly? The only way to be able to determine that sometimes is to look at statistics. And so, if you’re not allowing yourself to be counted because you’re afraid of being discriminated against, which is a true and valid fear, in a sense, you’re not empowering yourselves by saying, “I’m being counted, I’ve established a beachhead here, I’m here for you,” hopefully, in the way that we’re being here for you today, because you’ll be that island in the sea of otherwise whiteness that allows people to say, “I have a resource, I have a mentor, I have an advocate, or I have a sponsor.” So, there is risk involved, but I think you will benefit yourself and you’ll benefit a lot of people who come after you by having the courage to say, “yes, I am a minority and I’m proud of it!”

### How best to mentor the next generation of academic physicians

**Kenya Parks:** What can we do to better in the next generation of physicians as we mentor? How can we advance in a better way, because, given the statistics on individuals entering medical schools, in some ways, we’ve gone backwards. So as we usher in this new generation of physicians, what are our roles, what would you suggest that we do in our capacities to advance the cause?

**FM:** I think you have to tell people that they belong in medicine. We’re told in many ways that we don’t belong. You have to reinforce the idea that they do belong, that they bring with them the capacity to succeed. They bring with them the talent the healthcare system needs, and they bring with them the kinds of things that will then eventually make this country better. It’s that easy. In California, half of all the kids are Latino. If they’re not graduating from high school, they’re not going to college. In that scenario, what is the economic future for California? The Urban Institute has estimated that every child that lives in poverty costs the country $38,000. As a result of the number of children in poverty, we lose a half a trillion dollars per year. Think about what that says about our society. There are countries around the world poorer than ours that do better with their kids. So, to me, it makes economic sense to get the most out of everybody we have in the country. Moreover, we’ve got to help each other achieve this goal. When somebody stands up to act on this issue, you can’t just keep sitting down. You’ve got to stand up with them.

**EFA:** I would add that these things also impact who we hire as faculty members, who we hire as staff, who we bring on for residents and medical students, but none of us talking about the big picture in terms of the people we care for, and all of that is also important. If you are asked to serve on a search committee, you could react by saying that you don’t have time, but I would encourage you to consider it an opportunity. When you participate on a search committee, you have a voice in the selection of a leader or a colleague. That’s how we start changing institutions. Some of it is big picture, and you can have a significant impact if you’re a leader. Regardless of our title or our role, we have opportunities every day to think about and to advance these issues, but it will involve work, it will involve time, so it’s not going to happen just by sitting back and thinking that it’s important. It’s going to require that you invest in it, but I think that all of those ways ultimately can lead to institutional change.

## Conclusions

In conclusion, racial/ethnic minority young investigators in academic pediatrics attending the annual RAPID conference identified six hot topics on the most useful faculty and career-development issues for ensuring academic success and optimal career development for young clinician-investigators in academic medicine from diverse backgrounds. These six topics included: negotiating for protected research time, career trajectories as academic institutions move away from an emphasis on tenure-track positions, how “non-academic” products fit into career development, racism and discrimination in academic medicine and how to address them, coping with isolation as a minority faculty member, and how best to mentor the next generation of academic physicians.

Senior RAPID conference leadership provided responses to young minority investigators’ questions about these topics. Advice to young minority investigators on negotiating for protected research time includes identifying in advance what is specifically needed for success, understanding the priorities of the division chief, emphasizing rationale and return on investment, and demonstrating positive research outcomes with research effort provided to date. Key counsel on career trajectories as academic institutions move away from an emphasis on tenure-track positions includes demonstrating faculty contributions in all academic mission areas (clinical, education, scholarship, and service), understanding the changing nature of tenure, and comprehending how money flows at your institution. Guidance on how “non-academic” products fit into career development includes understanding the specific institutional criteria for advancement, appreciating the distinction between fulfilling institutional expectations and personal career goals, and the importance of op-eds in advancing advocacy.

Regarding racism and discrimination in academic medicine and how to address them, senior conference leadership recounted specific experiences with racism and gender discrimination, and advised reporting such incidents to the appropriate institutional leaders and offices, making sure to protect yourself emotionally when you are subjected to racism or discrimination, vigorously confronting racism and discrimination when they are encountered, and discussing and working through these issues with mentors and peers. On the subject of coping with isolation as a minority faculty member, senior conference leadership recommend identifying social support both within and across departments and schools at your institution, and being proud of one’s identity as a minority (rather than hiding it) in order to be a role model and resource. Concerning how best to mentor the next generation of academic physicians, senior conference leadership advise reinforcing for minority faculty a sense of belonging and the capacity to succeed, and taking on leadership opportunities that have the potential to advance the causes of diversity and institutional change for the better for minority faculty.

## Abbreviations

APA: Academic Pediatric Association
NIDDK: National Institute of Diabetes and Digestive and Kidney Diseases
NIH: National Institutes of Health
PAS: Pediatric Academic Societies
R01: NIH research project grant
RAPID: Research in Academic Pediatrics Initiative on Diversity
RFAs: Requests for applications
RVUs: Relative value units
URM: Underrepresented minority group
